# Open Questions in the Management of Nodular Lymphocyte Predominant Hodgkin Lymphoma

**DOI:** 10.1155/2014/427613

**Published:** 2014-02-20

**Authors:** Marguerite Tyran, Laurence Gonzague, Reda Bouabdallah, Michel Resbeut

**Affiliations:** Service de Radiothérapie Institut Paoli-Calmettes, 232 Boulevard Sainte Marguerite, 13009 Marseille, France

## Abstract

Localized Nodular Lymphocyte Predominant Hodgkin Lymphoma is a rare disease with an overall good prognosis but frequent late relapses. Due to it's rarity there is no standard therapeutic approach and pathological diagnosis may be hard. In this paper we discuss the technical aspects of the radiation therapy and histological issues. The new fields reductions proposed for classical Hodgkin lymphoma cannot be applied to early stages Nodular Lymphocyte Predominant Hodgkin lymphomas which are usually treated with radiation therapy without systemic chemotherapy.

## 1. Introduction

In this paper we describe two cases of failure in the management of nodular lymphocyte predominant Hodgkin lymphoma (NLPHL). Both treated outside our center, they were referred back to our unit for early relapse after “involved node” radiotherapy (INRT).

NLPHL is a rare disease (3–8% of all Hodgkin lymphomas) with distinct histology and clinical course. Despite frequent late relapses, NLPHL have paradoxically an overall good prognosis. Due to the rarity of the disease there is no standard therapeutic approach. In this short paper, we discuss the technical aspects of the radiation therapy and histological issues correlated with these two early relapses.

## 2. Case Presentation: First Case

The patient is a 53-year-old male who presented a submaxillary right tumefaction (IIA area).

The lymph node biopsy (June 2011) demonstrated NLPHL. The Ann Arbor classification was stage IA.

The PET scanner confirmed the unique uptake in the cervical area IIA.

**Figure 1:**
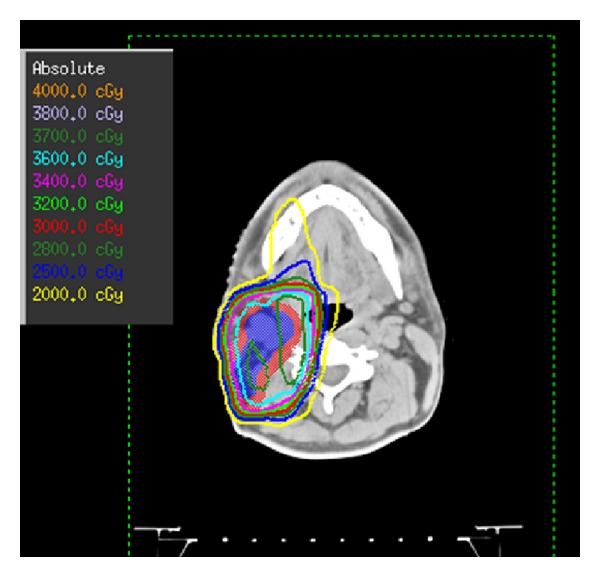
Dose distribution of a three-dimensional conformal radiotherapy: CTV (blue), PTV (red), and isodoses lines of 37 Gray (green), 36 Gray (cyan), 34 Gray (pink), and 20 Gray (yellow).

An INRT started in August 2011 and delivered 36 Gy within 18 fractions using conformational technique and was well tolerated (Figure 1).

**Figure 2:**
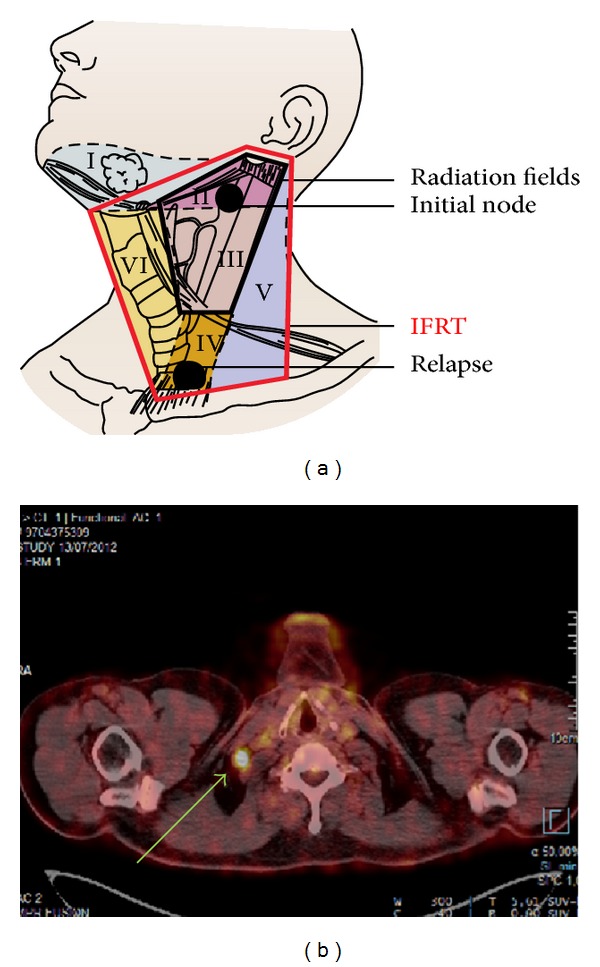
PET scanner showing the relapse in the subclavicular area and mapping.

During the followup the evaluation was assessed using PET scanners. The first PET scanner (January 2012) showed a complete response in the IIA area but a suspicious uptake in the subclavicular area (outside treated fields), confirmed as a relapse on the PET of July 2012 (Figure 2).

The biopsy of this early recurrence (5 months) demonstrated a relapse of NLPHL, confirmed in our hematological malignancies expert laboratory. The performance status (PS) of the patient was still 0; biology and clinical examination were normal.

## 3. Second Case

The patient is a 46-year-old male who presented a left cervical (III area) tumefaction.

After biopsy he was diagnosed with a NLPHL (October 2011) confirmed after a second analysis in an expert center outside our institution.

The Ann Arbor classification was stage IA. The initial PET scanner showed a unique left jugular-carotid fixation.

**Figure 3:**
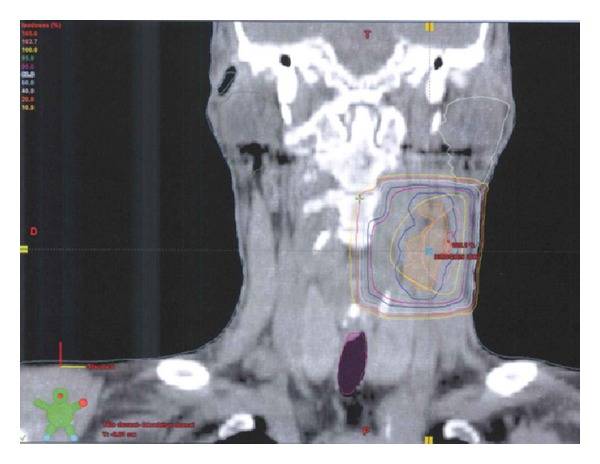
Dose distribution of a three-dimensional conformal radiotherapy: CTV (orange), PTV (blue), and isodoses lines of 105% (red), 100% (yellow), and 95% (green).

An INRT started in January 2012 and delivered 36 Gy within 18 fractions using conformational technique (Figure 3).

During the followup the evaluation was assessed using PET scanners.

**Figure 4:**
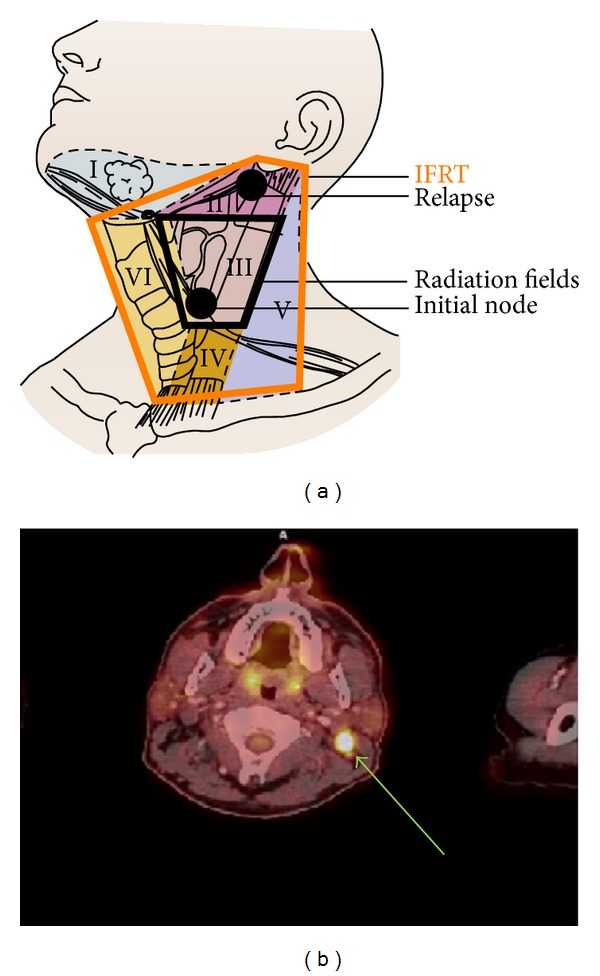
PET scanner showing the relapse in the IIB area and mapping.

The first PET scanner (October 2012) showed a unique FDG uptake in the IIB territory (outside treated fields), confirmed on a 2nd PET in November 2012 (Figure 4).

The biopsy of this early relapse (9 months after treatment) revealed a classical Hodgkin lymphoma (cHL). That unusual histological transformation led to a third analysis of the initial histological specimen in our expert center. The conclusion was in favor of a misdiagnosed lymphocyte-rich cHL, based on the positivity of CD30 and inconstant positivity of CD20.

## 4. Discussion

Those two cases of early failure (treated in 2 different centers) raise two problems in the management of NLPHL: first, the choice of the irradiation technique and, then, the difficulties of the histological diagnosis.

### 4.1. Natural History of the NLPHL Compared to cHL's

The natural history of NLPHL is very different from cHL's, and an extrapolation from one to another for both clinical and therapeutic features would be hazardous.

Nogová et al. [1] described in 2008 the clinical features of NLPHL compared to cHL: there is a male predominance with a median age at diagnosis of 30–40 years. Most patients present early stage disease (Ann Arbor stage I or II) and B symptoms are rare.

In this large GHSG studythe authors evaluated treatment outcomes for NLPHL versus cHL with a median followup of 50 months. The NLPHL patients experienced significantly better freedom-from-treatment failure (88% versus 82%; *P* = 0.0092) and overall survival (96% versus 92%; *P* = 0.016) which the authors attribute, in part, to the earlier stage at presentation of NLPHL.

Concerning the timing of the relapses, they reported less early relapse (0.8% versus 3.2%, *P* = 0.0037) and more late relapse (7.4% versus 4.7%, *P* = 0.0226) justifying a long followup.

Jackson et al. [2] confirmed the necessity of a long monitoring showing an even higher rate of recurrence compared to other studies (44%). These data could be explained by a longer followup than in the other series (30 years).

In our report the 2 relapses occurred far earlier than reported in literature (5 and 9 months). Median time to relapse (MTR) was 3–6 yrs in the review of the literature reported by Lee and LaCasce [3]. When these data are detailed, the MTR is reported to be over 3 years in most series [2, 4–6] and even more (10 years) for Wilder et al. [7].

To sum up NLPHL is an indolent disease with late relapses requiring a long followup.

### 4.2. Therapeutic Deescalation

The toxicity of treatments concerning both extended field RT (EFRT) and chemotherapy was studied in several papers [4, 5, 9–11]. In one study about cHL [12] there was more death reported in the group treated by chemoradiotherapy than those treated with chemotherapy alone.

Based on these observations, the management of NLPHL has progressively evolved to less aggressive modalities, as for the treatment of cHL [13, 14].

However, no randomized trial has been conducted comparing EFRT versus in-field radiotherapy (IFRT) among patients with favorable cHL. IFRT was adopted as the standard arm in many European trials (EORTC H7F, H8F, H9F, and GHSG HD10).

As previously mentioned, radiation therapy in the treatment of NLPHL has evolved from EFRT to IFRT [4, 6, 9, 10, 15].

A recent study (H10 EORTC/GELA) proposes a further reduction to INRT for cHL with a good response on a PET scanner after 2 courses of chemotherapy, selecting by this way patients with a good prognosis. Involved fields are described in a paper from Girinsky et al. [16].

The choice of that technique was based on the concept that chemotherapy is effective for microscopic disease. Therefore larger fields would not be necessary anymore.

Moreover the advent of the PET scanner enables a better identification of the pathological nodes.

The last justification was that radiations complications were related to the irradiated volume and total radiation dose [4, 5, 9, 10, 12].

Assuming similarities between cHL and NLPHL, some teams would apply that deescalation to the management of NLPHL, especially knowing that the prognosis was even better.

But there is no rational in the literature for using this “involved node” technique for the treatment of localized NLPHL without chemotherapy. The usual treatment is IFRT alone.

### 4.3. First Issue: The Choice of the Irradiation Technique

This technique (INRT) has probably been chosen in order to reduce toxicity in front of a good prognosis disease. For a cervical irradiation we care about salivary sparing even if the dose used for NLPHL does not induce late effects on the parotids. The dose commonly used is 30–36 Gy based on what is done for cHL, but no dose study exists for NLPHL.

Nevertheless INRT has never been described for NPLHL: it is an option for cHL treated with combined modalities.

In those 2 cases the use of INRT has led to early relapses in sites which would have been irradiated in an IFRT technique. Two aspects of these relapses are relevant.

Firstly the timing of the relapses is striking (5 and 9 months): it is far earlier than reported in literature [2–7]. This may be correlated to the fact that these 2 failures were very close to the initial disease.

Secondly their location would have been included in an in-field radiation (Figures 2 and 4). Thereby we can make the hypothesis that IFRT would have avoided such an evolution.

Indeed, most of the literature describes, when done, out-field recurrence after IFRT [4–7, 9] confirming the fact that with an optimal treatment as IFRT those 2 cases may not have experienced early relapse.

### 4.4. Second Issue: The Histological Diagnosis

We have reported in our 2nd case a revision of the histological initial diagnosis in light of an unusual anatomopathology of the relapse.

Transformation may occur in the natural history of NLPHL [17, 18]. But the transformation in cHL is rare. Most of the transformations observed were into large B cell lymphoma.

That outcome highlights the necessity of a new biopsy when a relapse occurs.

That also reminds us of the real complexity of pathological diagnosis in those border cases.

Distinction from cHL, especially from lymphocyte-rich cHL, has been shown to be a very common misdiagnosis (32% of errors in the European Task Force on Lymphoma study [19]). The diagnosis has to be reviewed in an expert center.

In our case the relapse was characterized by a lymphocytic proliferation with a nodular architecture. The atypical cells had a large nucleus and the immunophenotype responded CD4+, CD15−, CD21+, low CD20+, CD30+, Bcl6+. The reanalysis of the primary disease sample described atypical cells with large nucleus and lobulated nucleolus, CD30+, and inconstant CD20+.

Commonly NLPHL is characterized by the lobulated nature of the nucleus “popcorn” even if some cells may have large nucleoli as the Sternberg cells. If those last cells are too numerous, it raises the possibility of the diagnosis of lymphocyte-rich cHL [8, 20].

The immunophenotypic analysis usually respond CD45+; CD20+, CD79a+ (pan-B markers); bcl6+, CD10−, bcl2−, CD30−, CD15−, EBV−. On the opposite, a cHL would be CD30+, CD15+, CD20+ (for 40%), CD79a− and bcl6− (Table 1).

Our case does not fulfill every criterion; nevertheless the pathologists of the expert center concluded that it was a cHL.

## 5. Conclusion

There is no evidence for treating localized NLPHL with “involved node” radiotherapy. We have reported here 2 cases of early relapses for such treatment in areas which would have been included in IFRT.

The aim of this paper is to warn our community not to undertreat those patients who could be cured by optimal local treatment and to highlight once again the difficulty of the pathological diagnosis such as the necessity of biopsying again relapses and reanalyzing samples in expert centers when possible.

## Figures and Tables

**Table 1:** Histological features of NLPHL and cHL [8].

	NLPHL	cHL
Malignant cells	Nodular patterns of LP cells	Diffuse, interfollicular, and nodular Reed-Sternberg cells
Background appearance	Predominantly small B-lymphocytes	Reactive cellular infiltrate
CD30	−	+
CD15	−	+
CD45	+	−
CD79a	+	−
CD20	++	−
